# Risk management plans and drug safety in Canada: A cross-sectional study

**DOI:** 10.1177/09246479251414280

**Published:** 2025-12-28

**Authors:** Joel Lexchin

**Affiliations:** 1 School of Health Policy and Management, 7991 York University, Toronto, ON, Canada; 2 Department of Family and Community Medicine, University of Toronto, Toronto, ON, Canada

**Keywords:** drug safety, health Canada, risk management plan, transparency

## Abstract

**Background:**

Health Canada uses risk management plans (RMPs) to identify and monitor risks associated with drugs in the postmarket phase but does not make the contents of these RMPs public.

**Objective:**

To identify information about RMPs that is available in other Health Canada documents and to analyze extent of the information.

**Methods:**

A list of new active substances (NAS) approved by Health Canada between July 1, 2015 and May 5, 2025 was generated. These drugs were searched on the Summary Basis of Decision website and any information about the contents of the RMPs was copied verbatim. The information was read iteratively and grouped into themes.

**Results:**

There were 385 NAS approved and 381 had RMPs. For 301 the only information was that they had been reviewed by Health Canada and found to be acceptable. The information about the other 80 was typically vague and revealed little about how the information that they generated would be used to enhance safety.

**Conclusion:**

Health Canada needs to make RMPs publicly available, should explain how their implementation will be monitored and evaluated, how the information they generate will be used to enhance drug safety and should publish updates to them as necessary.

## Introduction

When a new active substance (NAS, a molecule never previously marketed) is approved, relatively little is known about its safety profile. This situation exists for a number of reasons including the population in the preclinical trials being relatively homogeneous^1^ and the rule of three in detecting side effects, that is, you need three times as many subjects to observe an event when you assume that the adverse event of interest does not normally occur in the absence of the medication. Therefore, if a side effect occurs one time in a thousand, three thousand patients have to be observed.^2^ The median number of patients enrolled in pivotal trials for drugs approved in Canada from 2012 to 2022 was 457^3^ meaning that safety problems occurring in fewer than 150 patients may have been missed.

In part, to help fill this knowledge gap about drug safety, since June 2015 Health Canada has adopted the use of risk management plans (RMP). Companies are expected to file RMPs when they submit an application to have a NAS approved if there is a significant degree of uncertainty respecting the risks associated with a particular drug or when a particular drug presents a serious risk of injury to human health that warrants measures to reduce the probability or severity of such an injury.^4^ However, the submission of a RMP until April 1, 2027 is voluntary on the part of the manufacturer.^5^ An RMP usually involves both pharmacovigilance and risk minimization activities. The former includes signal detection, the submission of regulatory safety summary reports, postmarket safety and utilization studies, clinical trials and patient registries. The latter are activities such as modifying the content of product labeling and the type of packaging, drug administration training, developing educational material for healthcare professionals and patients, restricting access and distribution and risk communication.^6^

According to Health Canada, “The main objective of the Risk Management Plan (RMP) is to identify how risks will be minimized and monitored to ensure that the benefit of a particular drug used in the treatment of patients outweigh the risks.”^7^ At the same time, the contents of RMPs are not made public, nor is there any information about how they are being used in practice to monitor and evaluate drug safety or when RMPs are updated. However, Health Canada does point to two sources of information about drug safety and RMPs^7^; the Drug Product Database,^8^ which “provides high-level information on additional risk minimization measures included in the product’s RMP”^7^ and the Summary Basis of Decision^9^ document that “includes information on the approved RMP, if any.”^7^

There has not been any previous analysis of Health Canada’s RMP system. The purpose of this study is to investigate the amount of information about RMPs in these two sources. Expanded knowledge about how RMPs work is necessary to understand how they can be used to enhance drug safety.

## Methods

### List of new active substances approved

A list of all NAS approved between July 1, 2015 and May 5, 2025 was generated from two sources. The first was the annual reports, until March 30, 2022, from the Therapeutic Products Directorate and the Biologics and Radiopharmaceutical Drugs Directorate, previously available from publications-publications@hc-sc.gc.ca. The second was the website maintained by Health Canada that lists completed drug submissions from December 2015.^10^ The generic and brand names of the drugs were recorded.

### Retrieval of information in RMPs

Each drug was searched on both the Drug Product Database^8^ and Summary Basis of Decision^9^ websites and any information about RMPs was recorded verbatim. RMPs requested by Health Canada once drugs were already marketed and for generics and biosimilars were not evaluated.

### Data analysis

Counts were made of the number of NAS with and without RMPs. The information collected about the contents of the RMPs was read iteratively and then categorized into themes and the number of times each theme was mentioned was recorded. (A single RMP could mention more than one theme.) No statistical analysis was undertaken.

### Patient involvement, ethics, and reporting

No patients were involved in this study, all data were publicly available and ethics approval was not required. Data were gathered by a single individual between May 10 and 15, 2025. The 2007 STROBE guidelines for cross-sectional studies were followed.^11^

## Results

**Figure 1.**
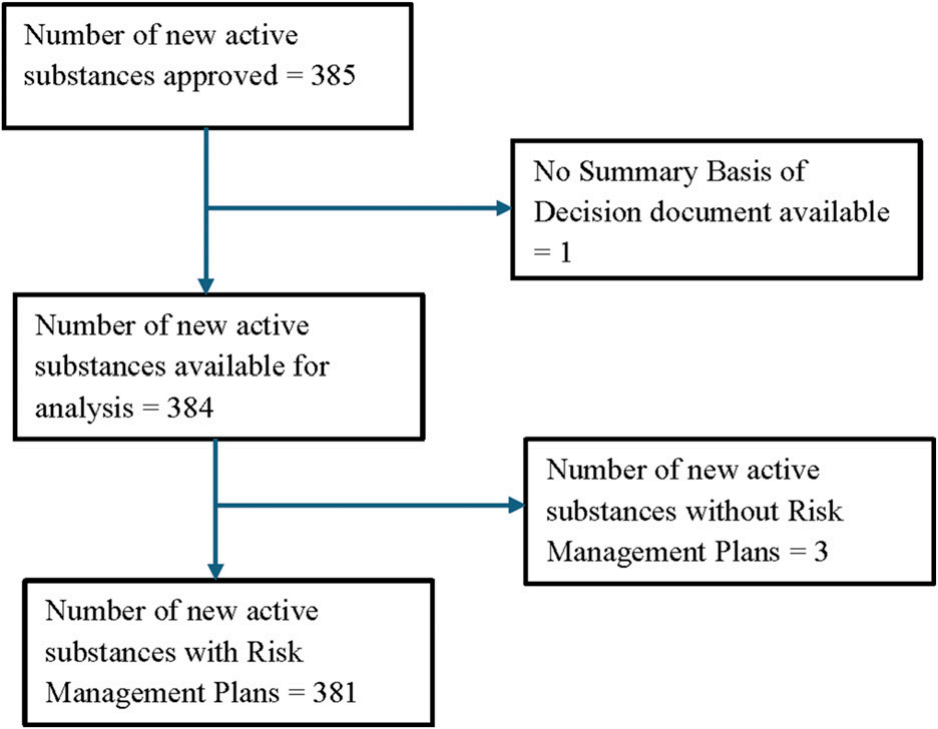
PRISMA flow diagram.

Three hundred and eight-five NAS were approved. One drug did not have an entry in the Summary Basis of Decision website and three drugs did not have a RMP leaving 381 NAS with a RMP for analysis (Figure 1). The only information on the Drug Product Database about RMPs was the statement that “A Risk Management Plan (RMP) for this product was submitted.” The only information in the Summary Basis of Decision about the RMP for 301 (79.0%) drugs was that the RMP was reviewed by Health Canada and found to be acceptable (Supplemental File 1).

The statements in the 80 (21.0%) RMPs with additional information or that mentioned that a RMP was not necessary were grouped into 9 themes with themes mentioned between 2 and 27 times, with a total of 95 mentions (Supplemental File 2). The most frequent theme was that the RMP needed to be updated, revised or corrected and the theme with the fewest mentions was instructions for initiating therapy (Table 1). In general, the themes reflected the types of pharmacovigilance and risk minimization activities that Health Canada outlined in its documentation about what RMPs should contain. However, the level of detail in the information about RMPs was typically vague as can be seen from the examples in Table 1. For example, the entry for peginterferon Beta-1A says that the company will carry out postmarket activities but there is no information about the nature of those activities. The entry for telotristat merely lists known risks incorporated in the RMP.

**Table 1.** Themes in information about Risk Management Plans.

Theme	Examples of information about content of risk management plans (drug name)	Number of risk management plans mentioning theme*
Update/revise/correct deficiencies in RMP	Provide an updated RMP† to reflect the Canadian labelling and the required postmarket activities to be carried out after authorization (peginterferon Beta-1A)	27
RMP includes important risks	Constipation and hepatic enzyme elevations are listed as important identified risks, while depression is considered an important potential risk (telotristat etiprate)	19
Conduct post-authorization studies/monitor risks	A post-authorization non-interventional clinical study will be conducted in Canada to document drug utilization, conversion rates, and safety in actual clinical settings (vernakalant)	17
Confirmation of adequacy of information in RMP	The safety profile is considered acceptable with the appropriate labeling and postmarket monitoring that has been put in place (cerliponase alfa)	9
Creation of educational material for healthcare professionals/patients	In addition, the sponsor was requested to provide educational material for healthcare professionals and patients (dinutuximab)	7
Submit regular periodic safety update reports/Periodic benefit-risk evaluation reports	Submit biennial periodic safety update reports/Periodic benefit-risk evaluation reports (PSURs/PBRERs) [about Tukysa] for the first 4 years following marketing in Canada; Include in the upcoming PSURs/PBRERs information specific to the summary of the safety data obtained from all pharmacovigilance activities (interim and final reports), and the ongoing/planned studies, including HER2CLIMB-02, an ongoing/planned randomized, double-blind, Phase 3 study of tucatinib or placebo… this study will enable further characterization of the safety profile for Tukysa and its long-term safety.	5
Undertake risk minimization activities	Risk minimization includes ongoing postmarketing surveillance, creation of a pan-Canadian Patient registry to evaluate the long-term safety and adequate labeling of all identified safety issues (nitisinone)	5
RMP not required or reasons why content of RMP is minimal	A risk management plan was not required or provided for this submission (gallium (68ga) chloride)	4
Instruction on initiation of therapy	Treatment should be initiated and supervised by a qualified physician experienced in the use of anticancer therapies (zanubrutinib)	2

*A single Risk Management Plan may contain more than 1 theme.

†RMP: Risk Management Plan.

Even when the RMP contained a greater amount of information, there were still significant deficiencies. For example, according to the RMP for tucatinib upcoming safety reports were to include safety data obtained from all pharmacovigilance activities and the ongoing/planned studies, including HER2CLIMB-02, an ongoing/planned randomized, double-blind, Phase 3 study of tucatinib or placebo. But there was no timeline attached to this commitment and no indication how this information would be used beyond that it will further enable the characterization of the drug and its long-term safety.

## Discussion

Virtually all the NAS approved by Health Canada have a RMP and since the submission of RMPs at the time of drug approval is still voluntary, it indicates that manufacturers seem to believe that there are significant unresolved safety issues associated with their drugs when they are approved. However, Health Canada provides almost no transparency about contents of those RMPs nor how they are used. Seventy-nine percent of the time, Health Canada merely says that the RMP was reviewed and found to be acceptable. The other 21% often just say that the RMP needs revising or updating, describes the risks included in the RMP and says that the manufacturer has committed to ill-defined postmarket studies or trials, although occasionally there is some concrete information.

In addition to problems with transparency there are also concerns about the design and administration of the RMPs. Both functions are carried out by manufacturers although Health Canada has to approve the RMPs. However, the commercial interests of companies may introduce biases in them, exemplified by the Suboxone Education Programme.^12^ The program, with a brand name in its title, was developed by Indivior, the corporate rights-holder for Suboxone, as part of a Health Canada regulated RMP. It is mandatory in some provinces to complete the program before healthcare professionals are able to prescribe buprenorphine-naloxone despite there being good, accredited, non-industry educational programs already available to teach appropriate opioid addiction care and prescribing. The inclusion of the brand name in the title flies in the face of the recommended practices and accreditation standards for Canadian medical education.^13^

Equivalents to the RMP system exist in other jurisdictions. The Food and Drug Administration uses its Risk Evaluation and Mitigation Strategies (REMS) program to identify and minimize risks in the United States in the postmarket period but is much more transparent about how REMS work. There is a public website that lists all the drugs with REMS and contains the goals of the individual REMS, a summary of the measures included in them, the materials that have been developed as part of the REMS and how the effectiveness of the REMS plan is going to be assessed.^14^ The European Medicines Agency also publishes the risk management plans that it has approved along with RMP updates.^15^

### Limitations

All the material was gathered by a single individual and that may have resulted in transcription errors although this problem was unlikely since information about the content of the RMPs was copied verbatim. The identification of themes also may have had a subjective element.

## Conclusion

Health Canada should make RMPs publicly available, should explain how their implementation will be monitored and evaluated, how the information they generate will be used to enhance drug safety and should publish updates to them. In addition, Health Canada should design the RMPs, with input from the drug companies, and have them administered by a neutral third party to avoid any commercial biases in them.

## Supplemental Material

Supplemental Material - Risk management plans and drug safety in Canada: A cross-sectional study Supplemental Material for Risk management plans and drug safety in Canada: A cross-sectional study by Joel Lexchin in International Journal of Risk & Safety in Medicine

Supplemental Material - Risk management plans and drug safety in Canada: A cross-sectional study Supplemental Material for Risk management plans and drug safety in Canada: A cross-sectional study by Joel Lexchin in International Journal of Risk & Safety in Medicine

## Data Availability

All the data collected for this study is in the two Supplementary Files.*
