# Supplementary material for: Risk management plans and drug safety in Canada: A cross-sectional study
Source: Int J Risk Saf Med. 2025 Dec 28;37(3):309–14. doi: 10.1177/09246479251414280 (PMC13428812; doi:10.1177/09246479251414280)
Supplement: Supplemental Material - Risk management plans and drug safety in Canada: A cross-sectional study [file sj-pdf-2-jrk-10.1177_09246479251414280.pdf]

Supplementary File 2: Information about contents of Risk Management Plans in Summary Basis of Decision

Generic name	Brand name	Contents of Risk Management Plan
Abrocitinib	Cibinqo	A Cibinqo Education Program comprised of educational materials for both healthcare providers and patients will be implemented by Pfizer Canada ULC. The program is part of the risk mitigation strategies specific to the potential risks of infections, thrombotic events including pulmonary embolism, malignancy, major adverse cardiovascular events, and embryo-fetal toxicity. The materials will include a Prescriber Brochure/Guide and Patient Card/Patient Safety Information Leaflet.
AD26.COV2.S	Janssen COVID-19 Vaccine	The RMP for the Janssen COVID-19 Vaccine includes information about the important potential risk of vaccine-associated enhanced disease (VAED) including vaccine-associated enhanced respiratory disease (VAERD). The RMP also identified seven areas of missing information: "use during pregnancy", "use in breastfeeding women", "use in immunocompromised patients", "use in patients with autoimmune or inflammatory disorders", "use in frail patients with comorbidities (e.g., chronic obstructive pulmonary disease, diabetes, chronic neurological disease, cardiovascular disorders)", "interaction with other vaccines", and "long-term safety". Upon review, Health Canada recommended the addition of additional important potential risks ("anaphylaxis" and "venous thromboembolism") and missing information ("use in the pediatric population <18 years of age" and "long-term effectiveness").
Amivantamab	Rybrevant	At the time of Notice of Compliance issuance, there were no RMP-related issues that would preclude the authorization of Rybrevant.
Andusomeran	Spikevax XBB.1.5	The RMP for Spikevax XBB.1.5 includes three important identified risks (anaphylaxis, myocarditis, and pericarditis). The RMP also identified four areas of missing information (limited/no clinical data): “use in pregnancy and while breastfeeding”, “long-term safety”, “long-term effectiveness”, and “use in subjects less than 18 years of age”. An important limitation of the data for all approved age groups continues to be the long-term safety and effectiveness of the vaccine.<br><br>There were no significant outstanding issues identified

		during the review of the safety data to preclude the authorization of Spikevax XBB.1.5. These will continue to be investigated through planned and ongoing studies, including Study mRNA-1273-P205 (Part J). Post-authorization commitments for monitoring the long-term safety and effectiveness of Spikevax XBB.1.5 have also been established. As outlined in the terms and conditions, the RMP will be updated to reflect additional safety information, including that which is relevant in a Canadian-specific context, as it becomes available. Results related to safety and effectiveness from ongoing and planned studies will be submitted as they become available.
Antihemophilic factor (recombinant, B-domain deleted, PEGylated)	Jivi	Health Canada requested inclusion of pediatric off-label use (for all children under 12 years of age) as an important potential risk in the next update of the RMP (accompanied with specific messages for educational materials to inform healthcare providers of the age restriction regarding the use of Jivi).
Avelumab	Bavencio	During the review, immune-mediated renal dysfunction, new-onset type 1 diabetes mellitus, and skin reactions were recategorized from potential risks to important identified risks of Bavencio.
Brolucizumab	Beovu	Health Canada recommended that the sponsor [of Beovu] update the RMP regarding important potential risks, risk minimization measures, medication/administration errors, systemic absorption and educational materials.
Cabotegravir sodium	Vocabria	The RMP [for Vocabria] includes drug resistance as an important identified risk, and hypersensitivity reactions and medication errors as important potential risks. These are expected to be monitored post-market.
Calasparagase pegol	Asparlas	As additional pharmacovigilance activities, the sponsor [of Asparlas] is expected to submit Periodic Safety Update Reports or Periodic Benefit-Risk Evaluation Reports every six months for the initial two years of marketing in Canada. The sponsor is also expected to provide a comprehensive safety review based on age group stratification and a cumulative review on reported cases with fatal outcomes with Asparlas, including those associated with off-label use.
Calcifediol	Rayaldee	A Risk Management Plan (RMP) for Rayaldee was not provided in this submission, as agreed upon with Health Canada, since the safety profile of calcifediol is well known. Rather, Vifor Fresenius Medical Care

		Renal Pharma Ltd. submitted an outline of their pharmacovigilance monitoring and reporting plan.
Caplacizumab	Cablivi	The sponsor [of Cablivi] is expected to submit an updated Canadian RMP to Health Canada prior to the launch of the product in Canada.
Cenegermin	Oxervate	In addition, to guide first-time users of the novel delivery system, the sponsor has developed a demonstration device kit and educational materials related to the Oxervate vial adapter and pipettes. The sponsor is expected to submit these materials for review by Health Canada prior to the commercial launch of Oxervate in Canada.
Cenobamate	Xcopri	The sponsor [of Xcopri] will monitor several identified and potential risks, including DRESS, QT interval shortening, suicidality, and reproductive and embryo-fetal toxicity. Furthermore, the sponsor will include, as an additional pharmacovigilance activity in the Canadian Addendum to the RMP, the following study required by the United States Food and Drug Administration: "A retrospective cohort study to assess the incidence of major congenital malformations in women with epilepsy exposed to cenobamate during pregnancy".
Cerliponase alfa	Brineura	Given the poor prognosis of this disease, the safety profile of Brineura is considered acceptable with the appropriate labelling and post-market monitoring that has been put in place through the Brineura Risk Management Plan (RMP).
Chadox1-S (recombinant)	Vaxzevria	Following Health Canada review, the RMP for the AstraZeneca COVID-19 Vaccine [Vaxzevria] includes the following as important potential risks: "anaphylaxis", "nervous system disorders including immune mediated neurological conditions", and "vaccine-associated enhanced disease including vaccine-associated enhanced respiratory disease". In addition, the following areas of missing information are included: "use in pregnant and breastfeeding women", "use in the pediatric population <18 years of age", "use in immunocompromised patients and patients with chronic or debilitating conditions", "use in subjects with severe and/or uncontrolled underlying disease", "interaction with other vaccines", "long-term effectiveness", and "long-term safety". It was noted that there was limited recruitment of persons ≥65 years of age and a lack of information regarding special populations (including Canadian indigenous

		populations, patients with chronic illness, immunocompromised individuals, and frail elderly) in the clinical data submitted by AstraZeneca Canada Inc.
Cilgavimab, tixagevimab	Evusheld	Upon review, the RMP was considered to be acceptable with revisions to include specific follow-up questionnaires for specific adverse reactions. The RMP included appropriate monitoring activities and risk minimization measures based on the known safety profile of Evusheld. The RMP will be updated to reflect additional safety information as it is collected.
Corynebacterium Diphtheriae CRM-197 Protein, Pneumococcal Polysaccharide Serotypes: 1, 3, 4, 5, 6A, 6B, 7F, 9V, 14, 18C, 19A, 19F, 22F, 23F, 33F	Vaxneuvance	At the time of Notice of Compliance issuance, there were no RMP-related issues that would preclude the authorization of Vaxneuvance.
Crisaborole	Eucrisa	Pfizer has initiated post-marketing clinical studies [for Eucrisa] supporting pediatric population.
Daclizumab beta	Zinbryta	A revised Canadian Risk Management Plan (RMP) that will:<br>• integrate the parameters/elements of the Biogen ONE Support Program and its associated registry;<br>• reflect the Canadian labelling for Zinbryta, the post-approval commitments to Health Canada and the international status.
Davesomeran, elasomeran	Spikevax Bivalent (Original / Omicron BA.4/5)	While clinical data for Spikevax Bivalent (Original / Omicron BA.4/5) are not yet available, based on extrapolation of clinical data from Spikevax Bivalent (Original/Omicron BA.1) and clinical and post-market data with the original Spikevax vaccine to date, including when used as a booster dose, the RMP for Spikevax Bivalent (Original / Omicron BA.4/5) includes three important identified risks (anaphylaxis, myocarditis, and pericarditis) and two important potential risks: vaccine-associated enhanced disease (VAED) and vaccine-associated enhanced respiratory disease (VAERD). The RMP also identified nine areas of missing (limited/no clinical data) information: “use in pregnancy”, “use in breastfeeding”, “long-term

		safety", use in immunocompromised patients", "use in frail patients with unstable health conditions and co-morbidities", "use in subjects with autoimmune or inflammatory disorders", "long-term effectiveness", "potential interaction with other vaccines and other drug products", and "use in subjects less than 18 years of age".
Dinutuximab	Unituxin	In addition, the sponsor [of Unituxin] was requested to provide educational material for healthcare professionals and patients.
Doravirine	Pifeltro	The RMP [for Pifeltro] was considered to be acceptable upon review, with revisions required within 90 days.
Drospirenone, estetrol	Nextstellis	In the RMP, VTE and ATE were considered important identified risks. The sponsor proposed routine pharmacovigilance activities and routine risk minimization measures (labelling) for these risks. The European Medicine Agency (EMA) requested an additional non-interventional post-authorization safety study (PASS) be carried out to further characterize the risk of VTE and ATE associated with Nextstellis in comparison with a marketed combined oral contraceptive. In this regard, Health Canada recommended that safety findings from post-marketing pharmacovigilance commitments, including the PASS required by the EMA, be submitted to Health Canada.
Elasomeran (mRNA)	Spikevax	The RMP for the Moderna COVID-19 Vaccine [Spikevax] includes information about the important potential risks of VAERD and anaphylactic reactions, including anaphylaxis. The RMP also identified six areas of missing (limited/no clinical data) information: "use in pediatric", "use in pregnant and breastfeeding women", "long-term safety", "long-term effectiveness" including "real-world use", "safety and immunogenicity in subjects with immune-suppression", and "concomitant administration with non-COVID vaccines". Upon review, Health Canada recommended that "younger than 18 years of age" be specified in the "use in pediatric" populations as missing information in the RMP. In addition, Health Canada recommended the addition of vaccine-associated enhanced disease (VAED) as an important potential risk.
Elotuzumab	Empliciti	In addition, as part of the marketing authorization for Empliciti, Health Canada requested and the sponsor

		agreed to several commitments to be addressed post-market. Commitments include (but are not limited to) providing an updated Canadian RMP in accordance with Health Canada's recommendations for additional risk minimization measures.
Estradiol, norethindrone acetate, relugolix	Myfembree	Additional pharmacovigilance activities consist of three ongoing post-authorization studies with Myfembree. Two studies (one observational study and the other a retrospective cohort study using an administrative healthcare database) will evaluate embryo-fetal toxicity in pregnant women and pregnancy outcomes. The third study (a single-arm, open-label study evaluating bone mineral density) will evaluate long-term use of Myfembree and the loss of bone mineral density.
Evinacumab	Evkeeza	Additional pharmacovigilance activities comprise two ongoing global post-authorization studies of Evkeeza. One study will evaluate the long-term safety outcomes in patients with homozygous familial hypercholesterolemia and the frequency and outcomes of pregnancy in female patients treated with Evkeeza. The second study is a descriptive pregnancy study aiming to evaluate pregnancy outcomes, maternal outcomes, and subsequent infant outcomes in women exposed to Evkeeza during pregnancy.
Famtozinameran, tozinameran	Comirnaty Original & Omicron BA.4/BA.5	While clinical data for Comirnaty Original & Omicron BA.4/BA.5 were not available at the time of authorization, the authorization was based on the extrapolation of clinical data from the bivalent Comirnaty Original/Omicron BA.1, clinical data obtained for the original Comirnaty vaccine used as a primary series and as a booster dose, as well as post-market safety data. The RMP for Comirnaty Original & Omicron BA.4/BA.5 includes three important identified risks (anaphylaxis, myocarditis, and pericarditis) and two important potential risks (vaccine-associated enhanced disease [VAED] and vaccine-associated enhanced respiratory disease [VAERD]). The RMP also identified eight areas of missing information (limited/no clinical data): "use during pregnancy and while breastfeeding"; "use in immunocompromised patients"; "use in frail patients with unstable health conditions and comorbidities"; "use in patients with autoimmune or inflammatory disorders"; "interaction with other vaccines"; "long-term safety"; "long-term vaccine effectiveness";

		and “use in the population less than 12 years of age”.
Ferric carboxymaltose	Ferinject	Upon review, the RMP [of Ferinject] was considered to be acceptable (with minor revisions to the Canadian-specific Addendum).
Ferric pyrophosphate citrate	Triferic AVNU	In the RMP, the sponsor [of Triferic AVNU] included ‘hypersensitivity reactions’ as an important identified risk; ‘systemic/serious infections’ as an important potential risk; and ‘use in pregnant and breastfeeding women’, ‘use in children’ and ‘concomitant use with other intravenous iron product’ as missing information. Labelling for these safety concerns has been included in the Product Monograph and the sponsor has committed to systemically review clinical and post-marketing safety data as part of routine pharmacovigilance activities.
Flibanserin	Addyi	In addition to the labelling in the Canadian Addyi Product Monograph, risk minimization measures include checklists for prescribers and pharmacists, as well as the distribution of the patient medication information at the time of dispensing. The objectives of these additional measures are to confirm the patients [sic] eligibility for treatment and to counsel patients for safe use of the medication including measures related to the increased risk of sedation and hypotension/syncope when the product is used with alcohol. Health Canada has requested that the sponsor conduct drug utilization studies to characterize real-world use of the product in Canada and to assess the effectiveness of the risk minimization measures.
Florbetaben 18F	NeuraCeq	…as part of the marketing authorization for NeuraCeq, Health Canada requested that the sponsor provide a reply for some follow-up points related to the RMP to be addressed post-market.
Fostemsavir tromethamine	Rukobia	At the time authorization, there were no RMP-related issues that would preclude the authorization of Rukobia.
Gallium (68ga) chloride	Galli Eo	A Risk Management Plan (RMP) was not required or provided for this submission, as Galli Eo does not have a clinical indication.
Gallium (68ga) chloride	GalliaPharm	The Risk Management Plan (RMP) provided for this submission was minimal, as GalliaPharm does not have a clinical indication.
Gallium (68ga) oxodotreotide	NETVision	An RMP for NETVision was not submitted by the Canadian Molecular Imaging Probe Consortium (CanProbe) to Health Canada. Health Canada agreed with the sponsor's position that an RMP was not

		necessary for several reasons. First, NETs are considered an orphan disease and the target population in Canada is relatively small. Second, consistent with most diagnostic positron emitting radiopharmaceuticals, this product presents inherently low risks to the target population. Based on the literature, ^{68}Ga-oxodotreotide has a well-characterized safety profile and all potential safety concerns are sufficiently controlled via drug product specifications or information in the Product Monograph. Finally, CanProbe operates under the Centre for Probe Development and Commercialization's (CPDC) existing Pharmacovigilance System. The CPDC has previously been found to be in compliance by Health Canada with respect to effectively monitoring for new safety signals for diagnostic PERs. The safety profile of NETVision will be monitored in accordance with this system and the applicable provisions of the Food and Drug Regulations.
Givosiran	Givlaari	The use of Givlaari in patients with moderate or severe hepatic impairment and in patients with end stage renal disease or on dialysis were included as missing information in the Risk Management Plan (RMP).
Icosapent ethyl	Vascepa	Educational materials will be made available to health care providers to reduce the risk of off-label use [of Vascepa].
Inclisiran	Leqvio	Upon review, Health Canada has required additional post-approval activities to be carried in order to ensure that the benefit of Leqvio continues to outweigh any risk after authorization of the drug.
Isatuximab	Sarclisa	Upon review for the RMP [for Sarclisa], several issues were identified by Health Canada. These issues were communicated to the sponsor and subsequently satisfactorily addressed by the sponsor.
Isavuconazonium sulfate	Cresemba	Upon review, the RMP [for Cresemba] was considered to be acceptable but requiring some revisions.
Metreleptin	Myalepta	Of note, there are four ongoing post-marketing safety studies [of Myalepta] which were required by the United States Food and Administration and the European Medicines Agency. The studies will further characterize the safety profile of metreleptin in patients with lipodystrophy. Given that lipodystrophy is a rare condition, the results will be globally applicable, and thus, also relevant to the Canadian setting.

Neratinib maleate	Nerlynx	At the time of NOC issuance, there were no RMP-related issues that would preclude the authorization of Nerlynx.
Nitisinone	MDK-nitisinone	Risk minimization includes ongoing post-marketing surveillance, creation of a Pan-Canadian Patient Registry to evaluate the long-term safety and adequate labelling of all identified safety issues, including the lack of data in some subpopulations, and the potential risk from use during pregnancy and lactation, along with the potential for risk to the fetus and mother from discontinuing [MDK-]nitisinone during pregnancy.
Nitisinone	Nitisinone	Risk minimization includes ongoing post-marketing surveillance, creation of a Pan-Canadian Patient Registry to evaluate the long-term safety and adequate labelling of all identified safety issues, including the lack of data in some subpopulations, and the potential risk from use during pregnancy and lactation, along with the potential for risk to the fetus and mother from discontinuing nitisinone during pregnancy.
Nitisinone	Orafin	Risk minimization measures include ongoing post-marketing surveillance, commitment to a Canadian post-approval safety study, and adequate labelling of all identified safety issues, including the lack of data in some subpopulations, the potential risk from use during pregnancy and lactation, and the potential risk to the fetus and mother from discontinuing nitisinone [Orafin] during pregnancy.
Ocrelizumab	Ocrevus	The sponsor has committed to provide, following approval, a revised Canadian RMP aligned with the revised Canadian Product Monograph information, applicable post-approval commitments to Health Canada, and updated international status of Ocrevus.
Odevixibat	Bylvay	The Sponsor [of Bylvay] will be requested to submit PSURs/PBRERs for review after 2 years and after 4 years following marketing in Canada, to allow further characterization of the safety profile associated with the use of the drug in the targeted population in the real-world setting. In addition, the Sponsor will be requested to submit interim/final results stemming from ongoing, planned, and post-market studies (including the ongoing open-label extension Study A4250-008, and the post-marketing studies requested by the United States Food and Drug Administration and by the European Medicines Agency for review by Health Canada.
Olipudase alfa	Xenpozyme	Of note, additional safety data will be provided from

		the ongoing DFI12712/ASCEND study, the long-term LTS13632 study, and a 5-year observational study (PMR 4291-1) that was required by the United States Food and Drug Administration to evaluate the long-term safety of Xenpozyme in pediatric patients under 2 years of age with acid sphingomyelinase deficiency and patients with acid sphingomyelinase deficiency type A.
Onasemnogene abeparvovec	Zolgensma	Upon review, Health Canada requested and the sponsor agreed to several commitments to be addressed post-market [for Zolgensma].
Panhematin	Hemin	Substantial deficiencies were identified in the RMP. Therefore, Health Canada requested that the sponsor submit a revised RMP within 90 days of the market authorization of Panhematin.
Peginterferon Beta-1A	Plegridy	As a post-approval commitment, the sponsor has been requested to provide an updated RMP [for Plegridy] to reflect the Canadian Labelling and the required post-market activities to be carried out after authorization.
Pneumococcal 20-valent Conjugate Vaccine	Prevnar-20	Of note, as part of additional pharmacovigilance activities, the sponsor is expected to provide results from an ongoing Phase IV, real-world study (Study B7471015) in the United States, designed to evaluate the effectiveness of Prevnar 20 against vaccine-type radiologically confirmed community-acquired pneumonia in adults 65 years of age and older. The sponsor is also expected to provide results from ongoing Phase III studies, including a safety and immunogenicity trial with Prevnar 20 co-administered with a seasonal inactivated influenza vaccine in adults 65 years of age and older, as well as results from pediatric trials to be evaluated for a potential extension of the indication to the pediatric population.
Pralatrexate	Folotyn	As part of the NOC/c Qualifying Notice [for Folotyn], the sponsor has been requested to provide any updates to the Canadian RMP when available.
Pralsetinib	Gavreto	At the time of authorization, there were no RMP-related issues that would preclude the authorization of Gavreto.
Prasterone	Intrarosa	Health Canada recommended that the safety specifications for Intrarosa be amended to include "abnormal Pap smear" as an important identified risk. The RMP is designed to describe known and potential safety issues, to present the monitoring scheme and when needed, to describe measures that will be put in place to minimize risks associated with the product.

Raxtozinameran	Comirnaty Omicron XBB.1.5	While clinical data for Comirnaty Omicron XBB.1.5 were not available at the time of authorization, based on the extrapolation of clinical and post-market safety data for the original Comirnaty vaccine, Comirnaty Original/Omicron BA.1, and Comirnaty Original & Omicron BA.4/BA.5 to date, the RMP adequately captured the known and potential risks of this vaccine. The RMP for Comirnaty Omicron XBB.1.5 includes three important identified risks (anaphylaxis, myocarditis, and pericarditis). The RMP also identified eight areas of missing information (limited/no clinical data): "use in pregnancy and while breastfeeding", "use in immunocompromised patients", "use in frail patients with comorbidities", "use in patients with autoimmune or inflammatory disorders", "interaction with other vaccines", "long-term safety", "vaccine effectiveness", and "use in pediatric individuals less than 6 months of age". An important limitation of the data for all approved age groups continues to be the long-term safety and effectiveness of the vaccine. This limitation is managed through labelling and the RMP.<br><br>Overall, the RMP was considered to be acceptable and identified appropriate monitoring (pharmacovigilance) activities and risk minimization measures for Comirnaty Omicron XBB.1.5 based on the known safety profiles of the original Comirnaty vaccine, Comirnaty Original/Omicron BA.1, and Comirnaty Original & Omicron BA.4/BA.5. This included providing information in the Comirnaty Omicron XBB.1.5 Product Monograph and identifying populations where more data are needed. Additional pharmacovigilance activities include continued safety surveillance using data collected from ongoing clinical and post-authorization studies undertaken for the currently authorized Comirnaty vaccines and the addition of two clinical studies (studies C4591048 and C4591054) evaluating the safety of Comirnaty Omicron XBB.1.5 vaccine in healthy individuals 12 years and older, and 6 months to less than 5 years of age. Two additional post-market studies are planned to evaluate the real-world effectiveness of the vaccine. The RMP will be updated to reflect additional safety information as this is collected.
Recombinant	Supemtek	The sponsor [of Supemtek] agreed to several

haemagglutinin protein-strain B(yamagata) + strain A(H1N1)+strain A(H3N2)+strain B(victoria)		commitments to be addressed post-market. Commitments include (but are not limited to) providing special analyses regarding anaphylactic reactions/hypersensitivity, pericarditis, Guillain-Barré Syndrome, neuritis, convulsion, encephalomyelitis/transverse myelitis, thrombocytopenia, syncope and vasculitis as Adverse Events of Special Interest (AESI) in clinical studies.
Reslizumab	Cinqair	In addition, as part of the marketing authorization for Cinqair, Health Canada requested that the sponsor agree to several commitments to be addressed post-market. Commitments include (but are not limited to) providing an updated Canadian RMP for Cinqair.
Riltozinameran, tozinameran	Comirnaty Original/Omicron BA.1	Based on the available data, the RMP for Comirnaty Original/Omicron BA.1 includes three important identified risks (anaphylaxis, myocarditis, and pericarditis) and two important potential risks (vaccine-associated enhanced disease [VAED] and vaccine-associated enhanced respiratory disease [VAERD]). The RMP also identified eight areas of missing information (limited/no clinical data): "use during pregnancy and while breastfeeding"; "use in immunocompromised patients"; "use in frail patients with unstable health conditions and comorbidities"; "use in patients with autoimmune or inflammatory disorders"; "interaction with other vaccines"; "long-term safety"; "long-term vaccine effectiveness"; and "use in the population less than 12 years of age".
Ritonavir, nirmatrelvir	Paxlovid	The sponsor is required to submit a Canadian RMP Addendum [for Paxlovid], which will address: use in patients with renal impairment; use in immunocompromised patients; use in pregnancy and breastfeeding; and drug/antiviral resistance and treatment-emergent mutations.
SARS-CoV-2 Recombinant Spike Protein	Nuvaxovid	The RMP for Nuvaxovid includes information about the important potential risks of vaccine associated enhanced disease (VAED) including vaccine associated enhanced respiratory disease (VAERD), anaphylaxis, and myocarditis and pericarditis. The RMP also identified eight areas of missing (limited/no clinical data) information: "use during pregnancy and while breastfeeding", "use in immunocompromised patients", "use in frail patients with comorbidities", "use in patients with autoimmune or inflammatory disorders", "interaction with other vaccines", "long-term safety", "use in pediatric subjects", and "long-

		term effectiveness".
SARS-COV-2 recombinant spike protein (Omicron XBB 1.5)	Nuvaxovid XBB.1.5	While clinical data for Nuvaxovid XBB.1.5 were not available at the time of authorization, the authorization was based on the extrapolation of clinical data for the original Nuvaxovid vaccine and the investigational Nuvaxovid platform vaccines targeting the Omicron BA.1 or BA.5 variant, and post-market safety data for the original Nuvaxovid vaccine to date. The RMP adequately captured the known and potential risks of this vaccine. This included two important identified risks: anaphylaxis, and myocarditis and pericarditis; and one important potential risk: vaccine-associated enhanced disease, including vaccine-associated enhanced respiratory disease. The RMP also listed eight areas of missing information (limited/no clinical data): “use in pregnancy and while breastfeeding”, “use in immunocompromised patients”, “use in frail patients with comorbidities”, “use in patients with autoimmune or inflammatory disorders”, “interaction with other vaccines”, “long-term safety”, “use in pediatric subjects”, and “long-term effectiveness”. An important limitation of the data for all approved age groups continues to be the long-term safety and effectiveness of the vaccine. This limitation is managed through labelling and the RMP.<br><br>Overall, the RMP was considered to be acceptable and identified appropriate monitoring (pharmacovigilance) activities and risk minimization measures for Nuvaxovid XBB.1.5 based on the safety profiles of the original Nuvaxovid vaccine and clinical data from the investigational Nuvaxovid platform vaccines targeting the Omicron BA.1 or BA.5 variant. This included providing information in the Nuvaxovid XBB.1.5 Product Monograph and identifying populations where more data are needed. Additional pharmacovigilance activities include continued safety surveillance using data collected from ongoing clinical and post-authorization studies. The RMP will be updated to reflect additional safety information as this is collected.<br><br>Terms and conditions have been imposed for the submission of Periodic Safety Update Reports/Periodic Benefit-Risk Evaluation Reports, RMPs, and post-authorization safety study reports to

		Health Canada, in addition to regulatory requirements for post-market monitoring. As mentioned in the terms and conditions, the RMP will be updated to reflect additional safety information, including that which is relevant in a Canadian-specific context, as it becomes available. Results related to safety and effectiveness from ongoing and planned studies will be submitted as they become available.
Sipavibart	Kavigale	For Kavigale, significant findings for the safety specifications section included the exclusion of pregnant patients in the clinical studies. Therefore, “Use in pregnancy” was the only important safety concern (missing information) that was listed in the RMP. No additional safety concerns were identified by Health Canada. Upon review, the RMP was considered to be acceptable.
Spesolimab	Spevigo	The sponsor will undertake additional and routine pharmacovigilance activities through the conduct of one post-authorization study and through the implementation of a questionnaire for serious or opportunistic infections (including sepsis) to further characterize the types of infections that could occur with Spevigo treatment, and a specific follow-up questionnaire for peripheral neuropathy.
Tafasitamab	Minjuvi	Upon review of the RMP [for Minjuvi], several issues were identified by Health Canada. These issues were communicated to the sponsor and were subsequently satisfactorily addressed by the sponsor.
Telotristat etiprate	Xermelo	Constipation and hepatic enzyme elevations are listed as important identified risks, while depression is considered an important potential risk in the Xermelo Risk Management Plan (RMP).
Tepotinib hydrochloride	Tepmetko	Appropriate warnings and precautions are in place in the approved Tepmetko Product Monograph to address the identified safety concerns, including a Serious Warnings and Precautions box describing the risk of hepatotoxicity, interstitial lung disease/pneumonitis, and embryo-fetal toxicity. In addition, treatment with Tepmetko should be initiated and supervised by a qualified physician experienced in the use of anticancer therapies.
Tibolone	Tibella	Upon review, the RMP [for Tibella] was considered unsatisfactory. A list of outstanding issues identified in the RMP were subsequently sent to the sponsor. The sponsor will revise the RMP to address these deficiencies and submit it for review.

Tirzepatide	Mounjaro	As part of the RMP [for Mounjaro], Eli Lilly Canada Inc. will conduct studies to further characterize the safety concerns of thyroid C-cell tumours, pancreatic malignancy, and diabetic retinopathy complications.
Tozinameran	Comirnaty	The RMP for the Pfizer-BioNTech COVID-19 Vaccine [Comirnaty] includes information about the important potential risks of VAERD. It also identifies missing (limited/not studied) information, to include use in pregnancy and lactation and vaccine effectiveness including "real-world" use. Upon review, Health Canada recommended the inclusion of long-term safety, use in immunocompromised patients and patients with chronic or debilitating conditions, and use in the pediatric population under 16 years of age as missing (limited/not studied) information.
Trastuzumab deruxtecan	Enhertu	As an additional risk minimization measure, in order to mitigate the risk of interstitial lung disease in the post-market setting, the sponsor [of Enhertu] proposed education materials for both healthcare professionals and patients, as well as a Patient Alert Card for patients. The purpose of the Patient Alert Card is to remind the patient of the key symptoms of lung problems that need to be reported immediately to healthcare professionals in order to facilitate early detection and treatment of interstitial lung disease.
Tremelimumab	Imjudo	At the time of Notice of Compliance issuance, there were no RMP-related issues that would preclude the authorization of Imjudo.
Tucatinib	Tukysa	The Sponsor will be requested to:<br>• Submit biennial Periodic Safety Update Reports/Periodic Benefit-Risk Evaluation Reports (PSURs/PBRERs) [about Tukysa] for the first 4 years following marketing in Canada;<br>• Include in the upcoming PSURs/PBRERs information specific to the summary of the safety data obtained from all pharmacovigilance activities (interim and final reports), and the ongoing/planned studies, including HER2CLIMB-02, an ongoing/planned randomized, double-blind, Phase 3 study of tucatinib or placebo in combination with T-DM1 for patients with unresectable locally-advanced or metastatic HER2-positive breast cancer. This study will enable further characterization of the safety profile for Tukysa and its long-term safety.
Upadacitinib	Rinvoq	As an additional risk minimization measure, the

		sponsor intends to distribute educational materials to inform healthcare professionals and patients of the risks of tuberculosis, herpes zoster, foetal malformation (pregnancy risk), and major adverse cardiovascular events, which have been associated with Rinvoq.
Vernakalant	Brinavess	To ensure appropriate determination of patient eligibility to receive treatment, and awareness of infusion monitoring and administration of Brinavess, the use of a pre-infusion checklist by health care professionals was implemented as part of the RMP. In addition, a post-authorization non-interventional clinical study will be conducted in Canada to document drug utilization, conversion rates, and safety in actual clinical settings.
Virus-like particles of SARS-CoV-2 Spike Protein	Covifenz	The RMP for Covifenz includes information about the important potential risks of anaphylaxis and severe allergic reactions, vaccine-associated enhanced disease (VAED), including vaccine-associated enhanced respiratory disease (VAERD) and pIMDs. The RMP also identified seven areas of missing (limited/no clinical data) information: "use in pregnant and breastfeeding women", "use in pediatric population", "use in immunocompromised subjects", "use in subjects with autoimmune or inflammatory disorders", "use in frail subjects with unstable health conditions and comorbidities", "interaction with other vaccines", and "long-term safety". Upon review, Health Canada recommended the inclusion of "long-term effectiveness" as missing (limited/no clinical data) information.
von Willebrand factor (recombinant)	Vonvendi	The RMP is considered acceptable, with updates expected within 90 days of receiving marketing authorization for Vonvendi.
Vorasidenib citrate	Voranigo	Servier Canada Inc. will be requested to submit Periodic Safety Update Reports (PSURs)/Periodic Benefit-Risk Evaluation Reports (PBRERs) for review after 2 years and 4 years of marketing in Canada, and to discuss the following safety topics as topics with special interest: long-term safety, ototoxicity, and safety in the pediatric/adolescent patient population (12 to less than 18 years) including growth and development [for Voranigo].
Zanubrutinib	Brukinsa	A Serious Warnings and Precautions box has been added to the Brukinsa Product Monograph. It includes the instruction that treatment with Brukinsa should be

		initiated and supervised by a qualified physician experienced in the use of anticancer therapies, and highlights the risk of serious hemorrhage.
